# Deficiency of a peroxisomal NADP-isocitrate dehydrogenase leads to dwarf plant and defect seed in upland cotton

**DOI:** 10.3389/fpls.2022.1000883

**Published:** 2022-09-14

**Authors:** Yuefen Cao, Wanru Wang, Jinhong Chen, Shuijin Zhu, Tianlun Zhao

**Affiliations:** ^1^College of Agriculture and Biotechnology, Zhejiang University, Hangzhou, China; ^2^Zhejiang Key Laboratory of Crop Germplasm, Zhejiang University, Hangzhou, China; ^3^Hainan Institute, Zhejiang University, Sanya, China

**Keywords:** perICDH, *Gossypium*, plant height, seed development, subcellular localization

## Abstract

The NADP-isocitrate dehydrogenase-encoded gene *GH_D13G1452* with a C-terminus tripeptide Proline-Lysine-Leucine was localized in the peroxisome. It was highly expressed in stems and ovules of 15 days post-anthesis and responded to multiple external stimuli in upland cotton. An upland cotton mutant (*Ghpericdh*) was identified by flanking sequence amplification and genome variation detection that exogenous sequence was inserted in the middle of the 12th intron of *GH_D13G1452*, resulting in the deficiency of gene expression. The *Ghpericdh* mutant displayed a dwarf plant phenotype when grown under field or greenhouse conditions, and *GH_D13G1452* functioned as an incomplete dominance on plant height. The germination rate of mutant seed from greenhouse-grown plants was dramatically lower than that from field-grown plants, which indicated that GhperICDH plays a critical role in seed maturation and germination. Therefore, *GH_D13G1452* is indispensable in the development of stems and seeds and functions in the adaptability of cotton to the environment. The *Ghpericdh* mutant provides insight into the function of peroxisomal ICDH and may contribute to the genetic improvement in cotton.

## Introduction

NADP-dependent isocitrate dehydrogenase (ICDH, EC. 1.1.1.42) catalyzes the oxidative decarboxylation of isocitrate to form 2-oxoglutarate and NADPH, which are involved integrally in ammonia assimilation and reactive oxygen species metabolism (Hodges et al., 2003). Through subcellular localization, various ICDH isozymes were identified, such as cytICDH localized in the cytoplasm, mitICDH in the mitochondrion, chlICDH in the chloroplast, and perICDH in the peroxisome, of which the activity of cytICDH accounts for more than 90% of overall isozymes (Mhamdi et al., 2010). In Arabidopsis, deficiency of cytICDH or perICDH does not affect plant growth under normal conditions (Mhamdi et al., 2010; Mhamdi and Noctor, 2015). Deletion of perICDH results in stomata semi-closed without a detrimental effect on the overall ICDH activity and redox homeostasis in Arabidopsis (Mhamdi and Noctor, 2015; Leterrier et al., 2016). These studies in Arabidopsis suggested that perICDH was not necessarily involved in plant growth, which can be explained by 2-oxoglutarate which is mainly produced by mitochondrial NAP-dependent isocitrate dehydrogenase (EC. 1.1.1.41) (Behal and Oliver, 1998), and the NADPH could be compensated by the pentose-phosphate pathway and NADH phosphorylation in plants (Corpas et al., 1998; Waller et al., 2010).

Here, we identified a cotton peroxisomal ICDH (GhperICDH) that was highly expressed in stems and seeds, and deficiency of *GhperICDH* would lead to abnormal plant growth and seed development in upland cotton.

## Materials and methods

### Plant materials

*Gossypium hirsutum* cv. TM-1 and mutant *Ghpericdh* were used here. TM-1 is a standard genetic line of upland cotton, obtained from USDA-ARS, College Station, TX, United States. The *Ghpericdh* is a deficiency mutant of the *GH_D13G1452* gene, generated from CCRI49 as a receptor overexpressing a glyphosate resistance gene *g10evo* (Tan, 2016), which was developed by our lab for five generations, and its wild-type (non-1007), a non-transgenic line with normal *GH_D13G1452* separated from the selfing transgenic plant T0, were used in the experiment. CCRI49 is a conventional cotton cultivar, which was provided by the Cotton Research Institute, Chinese Academy of Agricultural Sciences. All cotton plants were grown in the Agricultural Station field from May to September (Zhejiang University, Hangzhou) or in a greenhouse at 28^°^C/25^°^C under a 14-h photoperiod with a light intensity of 35000 lx.

### DNA and RNA extraction and PCR

DNA and total RNA were extracted from fresh young leaves. cDNA synthesis, RT-PCR, and qPCR were performed according to Cao et al. (2021). Fusion primer and nested integrated PCR (FPNI-PCR), used for flanking sequence amplification, were designed according to methods described previously (Li, 2016; Xu, 2017). Primers in this study are listed in Supplementary Table 1.

### Southern blot

The genomic DNA of 30 μg was digested completely with Hinde, separated by 0.8% gel electrophoresis, transferred into a nylon membrane (Amersham, United Kingdom), and hybridized with digoxin-labeled DNA fragments of *g10evo* at 65^°^C overnight. The signaling was detected by the image analyzer FLA-5100 (FUJIFILM, Japan). Detailed procedures were as described in the DIG High Prime DNA Labeling and Detection Starter Kit II (Roche, Switzerland).

### Subcellular localization

Full-length open-reading frames (ORFs) of *GH_D13G1452* fused with a superfolder green fluorescent protein (sGFP) on its N-terminus or C-terminus and driven by CaMV35S (pCAMBIA1300 vector), transiently co-expressed with the known peroxisomal markers 984 fused with mCherry in epidermal cells of tobacco *via* Agrobacterium (Nelson et al., 2007). The GFP and mCherry fluorescence in epidermal cells of tobacco was detected and photographed by a laser confocal microscope (Olympus, Japan) after injection from 48 to 72 h.

### Vector construction and genetic transformation in cotton

The complete cDNA of gene *g10evo* (Tan, 2016), amplified from *Deinococcus Radiodurans*, was ligated into the overexpression vectors pCAMBIA-1300 (driven by the CaMV35s promoter) and transformed into *Gossypium hirsutum* cv. CCRI49 according to methods described previously (Yan, 2011). The transformants were selected on a selective medium containing 2.0 M glyphosate.

### Stress treatment

The TM-1 seedlings were transferred into a plastic bucket of 1 L with a 1/2 MS culture medium. When growing to a two-leaf stage, plants were stressed by 10^–4^ M IAA, 10^–5^ M GA, 10^–7^ M ABA, 10^–4^ M JA, 10^–3^ M SA, 15% PEG6000, and 0.1-M NaCl for 3, 6, and 24 h with the non-treatment group as control. The roots were used for extracting RNA. The tendency (T) of the gene relative expression level over time under different treatments was calculated as *T* = 2^–(Δ*Ct*)^ of the treatment group - 2^–(ΔCt)^ of the control group.

### Measurement for plant height and germination rate

The plant height was measured from the base of hypocotyl to the first fully expanded true leaf. Here, the cotyledon is defined as the first node, the first leaf of the main stem is the second node, and so on. The distance between two adjacent leaves is defined as internode length. Internode length = Plant height/node numbers (each line repeated 6 individual plants). The germination rate was computed as the proportion of the germinated seeds in 15 days to the total seeds used (three replicates with 50 seeds in each one). Seed germination was carried out under a greenhouse at 28^°^C/25^°^C under a 14-h photoperiod with a light intensity of 35,000 lx.

### Measurement for stomatal aperture

The experiment was started in the morning after 10 h of the dark cycle. Cotyledon detached from cotyledon-stage seedlings was floated on the incubation medium (10-mM MES, 50-mM KCl, 100-μM CaCl_2_, pH = 6.1) in Petri dishes under light for 2 h to make stomata open fully (Xi et al., 2019). The lower epidermis was peeled off, transferred to a drop of incubation medium on a glass slide, and immediately observed under a microscope (Nikon Eclipse Ni, Japan) for stomata and photographed. The long axis and short axis of the stomatal aperture were measured to calculate the opening degree (OD). OD = short axis/long axis. Three individuals were randomly selected from each genotype, and three visual fields were randomly selected from each cotyledon.

### Sequence analysis and phylogenetic tree construction

Pairwise sequence comparisons were conducted using ClustalW (Thompson et al., 1994). Sequence similarities were analyzed with BioEdit (Tippmann, 2004). The phylogenetic tree was constructed by the neighbor-joining method with a bootstrap replication of 500 by using MEAG 5 (Tamura et al., 2011).

### Genome variation detection

Whole-genome resequencing of the *Ghpericdh* mutant was carried out with Oxford Nanopore Technologies (ONT) by Novogene (Tianjin, China) (Supplementary Table 2). Based on the resequencing data, the reads that could be mapped to both the *g10evo* gene and the cotton genome were extracted with NextGenMap-LR software (Sedlazeck et al., 2018). Then, the BAM files were obtained by comparing the extracted reads with the cotton genome. The VCR files were obtained by detecting the structural variation of the extracted BAM files using sniffles software (Sedlazeck et al., 2018). Based on the results of variation detection, the insertion sequence was extracted and compared with the *g10evo* gene for similarity analysis.

## Results

### Identification for a perICDH in upland cotton

**FIGURE 1:**
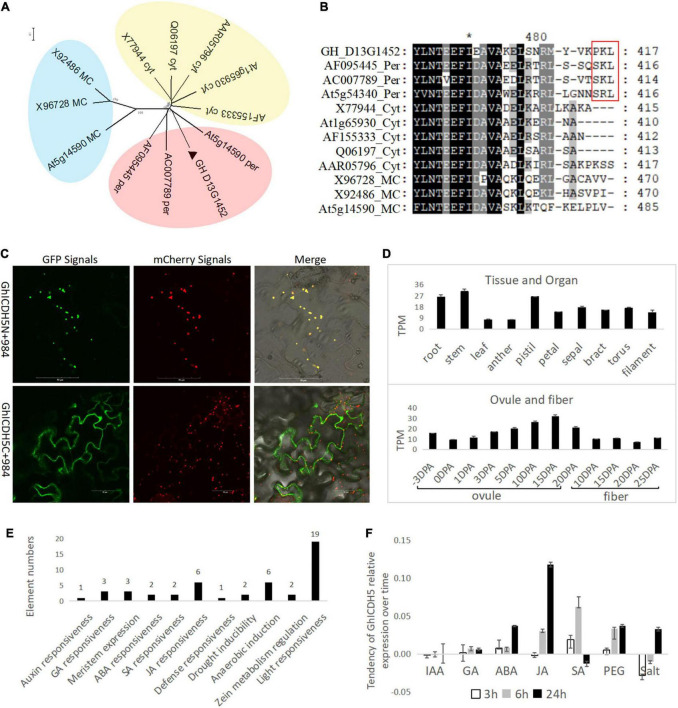
Identification for a perICDH in upland cotton. **(A)** Phylogenetic analysis of GH_D13G1452 (labeled by ▲) with ICDHs from other species (information, see Table 1) by the neighbor-joining method with a bootstrap replication of 500 based on amino acid sequences. All of proteins could be divided into three groups labeled by different colors. The peroxisomal ICDH group was filled with red; the cytoplastic ICDH group, yellow; the mitochondrion/chloroplast ICDH group, blue. **(B)** Sequence comparison of the putative ICDHs from different species. The type-I peroxisomal targeting signal (PTS1) in C terminus of peroxisomal proteins (labeled by the red box). Per, peroxisome; Cyt, cytoplasm; MC, Mitochondrion/chloroplast. **(C)** Subcellular localization. ICDHs fused with GFP at their C/N-terminus transiently co-expressed with the peroxisomal marker fused with mCherry in epidermal cells of tobacco. Nine hundred eighty-four, a peroxisome localization marker fused with mCherry. C, C-terminus-fused GFP; N, N-terminus-fused GFP. Scale bar = 50 μm. **(D)** Gene expression in different organs and at different stages of ovule and fiber development. DPA, day post-anthesis. **(E)** *Cis*-elements related to environmental response in the promoter of *GH_D13G1452*. **(F)** The response of *GH_D13G1452* to different abiotic stresses.

GH_D13G1452 has a high similarity of 84.4% with the peroxisomal ICDH from soybeans in the amino acid sequence (Table 1) and belongs to the peroxisomal ICDH group based on phylogenetic analysis (Figure 1A). The peroxisomal ICDHs possess the type-I peroxisomal targeting signal (PTS1), a tripeptide sequence typically found at the C terminus of peroxisomal proteins (Gould et al., 1989), such as SKL existed in perICDHs of soybeans and SRL in Arabidopsis perICDH, while Proline-Lysine-Leucine (PKL) was observed in the C-terminal of GH_D13G1452 (Figure 1B). Subcellular localization further confirmed that the peroxisomal targeting signal was located at the C-terminus of GH_D13G1452, while the peroxisomal signals disappeared when their C-terminus fused GFP (Figure 1C). Based on the above results, we named *GH_D13G1452* as *GhperICDH*.

**TABLE 1:** The similarity of amino acid sequences of GH_D13G1452 to other plants’ ICDHs.

Accession number	Host organism	Subcellular localization	Amino acid identity (%)	References
AF095445	Soybean	Peroxisome	84.4%	Hodges et al., 2003
AF155333	Rice	Cytoplasm	83.5%	Hodges et al., 2003
At5g54340	Arabidopsis	Peroxisome	82.5%	Leterrier et al., 2016
X77944	N. tabacum	Cytoplasm	82.5%	Hodges et al., 2003
AC007789	Rice	Peroxisome	82.2%	Hodges et al., 2003
Q06197	Soybean	Cytoplasm	81.5%	Hodges et al., 2003
At1g65930	Arabidopsis	Cytoplasm	81.3%	Leterrier et al., 2016
AAR05796	Poplar	Cytoplasm	81.3%	Pascual et al., 2018
X96728	N. tabacum	Mitochondrion/chloroplast	65.8%	Hodges et al., 2003
X92486	Potato	Mitochondrion/chloroplast	64.6%	Hodges et al., 2003
At5g14590	Arabidopsis	Mitochondrion/chloroplast	64.2%	Leterrier et al., 2016

Transcripts Per Million (TPM) data of upland cotton transcriptome (Hu et al., 2019) downloaded from CottonFGD were used to analyze the gene expression pattern in different tissues and organs. The *GhperICDH* was expressed in different tissues and organs with a peak level in stem (30.39 TPM) and had variable levels at different stages of the ovule and developed fibers with peak level (31.7 TPM) in ovules of 15 days post-anthesis (DPA) (Figure 1D). *Cis-*elements analysis showed that a large number of environmental response elements are found in the 3-kb region upstream of *GhperICDH*, besides the core elements of the promoter (Figure 1E), which indicates that *GhperICDH* responds to a variety of stress conditions. Treating with IAA, GA, ABA, JA, SA, PEG, and salt for 3, 6, and 24 h, the expression trend of *GhperICDH* showed that this gene had obvious responses to ABA, JA, SA, PEG, and salt (Figure 1F).

### Identification of a *GhperICDH*-deficiency mutant

**FIGURE 2:**
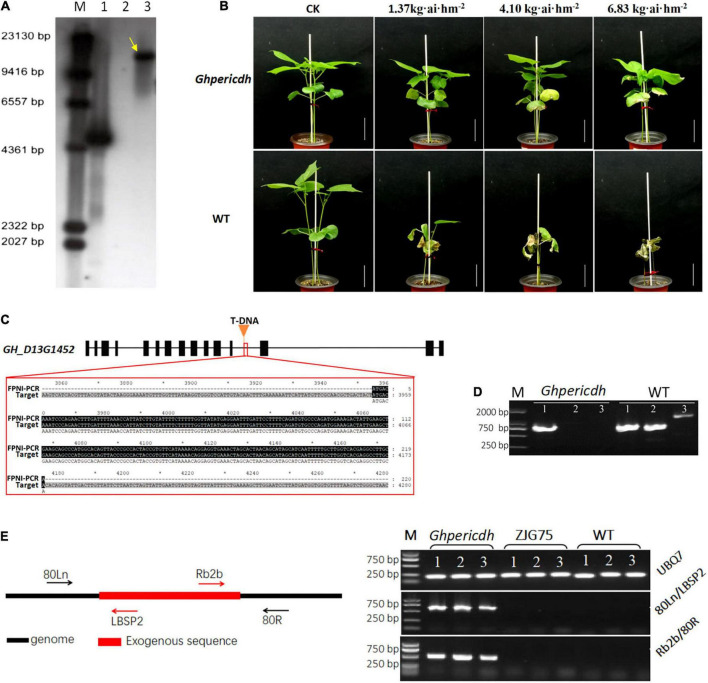
Identification of mutant *Ghpericdh*. **(A)** Southern blotting with a *g10evo* probe. The yellow arrow displayed one copy of the glyphosate resistance gene in the *Ghpericdh*. M, marker; 1, positive plasmid; 2, Wild-type plants; 3, *Ghpericdh* plants. **(B)** Glyphosate resistance of *Ghpericdh*. Scale bar = 5 cm. **(C)** The insertion position of *g10evo* in *GH_D13G1452*. The orange triangle represents the inserted position. The red box showed the flanking sequence (the black region) from FPNI-PCR. **(D)** RT-PCR analysis of *GH_D13G1452*. M, marker; 1, the partial cDNA fragment of homologous gene *GH_A13G1507*; 2, the partial cDNA fragment of *GH_D13G1452*; 3, complete cDNA of *GH_D13G1452.* **(E)** Specific molecular markers for mutant *Ghpericdh*. The left is the positions of two groups of primers 80Ln/LBSP2 and Rb2b/80R. The right was the amplification results with 80Ln/LBSP2 and Rb2b/80R in *Ghpericdh* and other lines. ZJG75, another transgenic glyphosate-resistant cotton line. UBQ7 was used as positive control. 1, 2, and 3 represent three individual plants.

A *GhperICDH*-deficiency mutant with glyphosate resistance was identified, and we named it *Ghpericdh.* Southern blotting displayed one copy of *g10evo* in the mutant (Figure 2A). *Ghpericdh* plants could grow normally under the recommended concentration of glyphosate isopropylamine in a field (1.37 kg⋅ai⋅hm^–2^), while became damaged and displayed leaf malformation under high concentrations (4.10 and 6.83 kg⋅ai⋅hm^–2^). Wild-type (WT) plants died under any concentration of glyphosate isopropylamine (Figure 2B). FPNI-PCR revealed the accurate position of *g10evo*, which was located in the middle of the 12th intron of *GH_D13G1452* (Hu et al., 2019; Figure 2C). Genome variation detection of the mutant found only one site on 45,469,231 nt of chromosome D13 of *G. hirsutum cv*. TM-1 genome containing an insertion sequence that possessed a high similarity of 92.4% with the *g10evo* gene, which was consistent with the result of FPNI-PCR. *GH_D13G1452* did not express in mutant *Ghpericdh* but did express in WT (Figure 2D). As controls, the homologous gene *GH_A13G1507* and the housekeeping gene *GhUBQ7* did normally express in both mutant and WT (Figure 2D and Supplementary Figure 1). Based on the genome information of mutant *Ghpericdh*, two specific molecular markers 80Ln/LBSP2 and Rb2b/80R only amplified in *Ghpericdh* were designed (Figure 2E and Supplementary Table 1).

### Phenotypic characteristics of *Ghpericdh* mutant

**FIGURE 3:**
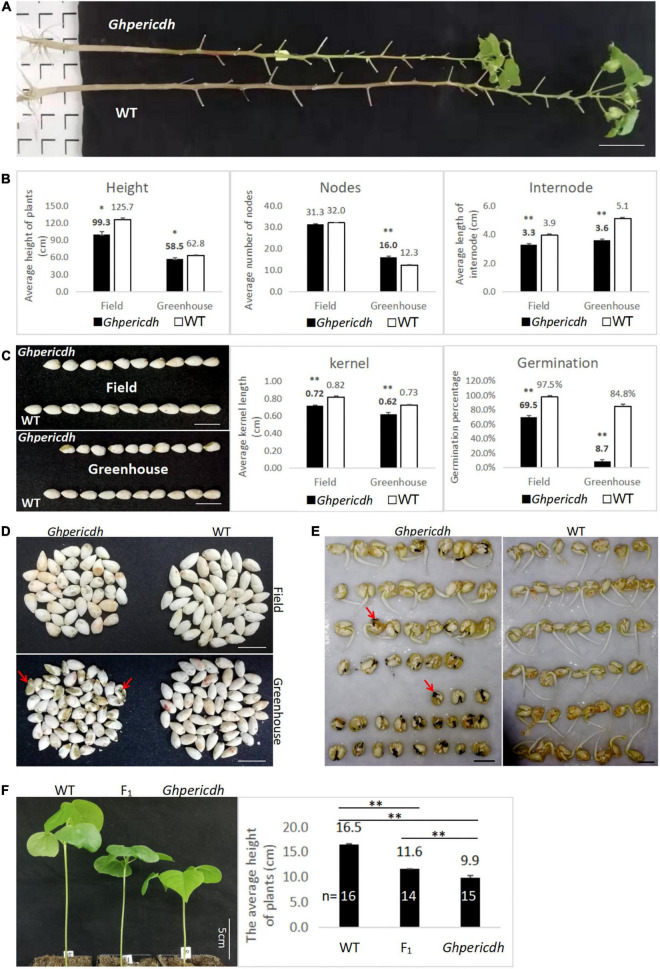
Phenotypic characteristics of *Ghpericdh*. **(A)** Plants growing for 5 months in the field. Scale bar = 10 cm. **(B)** Comparison of plant height, node number, and internode length between genotypes in the field for 5-month growing and in the greenhouse for 3 months cultured. **(C)** The kernel length and the germination rate of seeds harvested from the field and the greenhouse. Scale bar = 1 cm. **(D)** The kernel characteristics harvested in different locations. Scale bar = 1 cm. **(E)** Comparison of greenhouse-seed germination between genotypes. Scale bar = 1 cm. **(F)** Seedling morphology (the left) and average plant height (the right) of F_1_. *p < 0.05, ^**^p < 0.01.

The mutant *Ghpericdh* displays a dwarf phenotype (Figure 3A), but it can grow, flower, and bear fruits. Whether the plants growing naturally in the field or cultured in the greenhouse, the plant height and the internode length of mutants were significantly lower than those of WT (Figure 3B). The seeds from the mutant plants were shorter than WT seeds (Figure 3C). The greenhouse condition caused a decline in seed size of the mutant, which kept a similar trend in the WT as well (Figure 3C). Interestingly, the germination rate of seeds from greenhouse-grown mutant plants was dramatically lower than that from field-grown plants, i.e., 8.7 vs. 69.5%. In comparison, the seed germination rate from WT only decreased from 97.5 to 84.8% (Figure 3C). An observation from the *Ghpericdh* kernel found that seeds from the greenhouse appeared black (Figure 3D, marked by the red arrow) that became necrotic gradually during germination (Figure 3E), which led to growth stagnation. Similar to the Arabidopsis *pericdh* mutant, most stomata of *Ghpericdh* kept semi-closed (OD = 0.25-0.5) under light, while those of WT stayed open (OD ≧0.5) (Supplementary Figure 2).

F_1_ heterozygotes were obtained from the reciprocal crosses between *Ghpericdh* and WT. The plant height of F_1_ plants at the two-leaf stage was 11.6 cm between *Ghpericdh* (9.9 cm) and WT (16.5 cm) (Figure 3F). Among the 83 F_2_ plants from the self-pollinated F_1_ plant, the plant numbers of mutant/heterozygous/WT phenotype of plant height were 19/39/25, respectively, that the segregation conformed to 1:2:1 (χ^2^ = 1.17, *p* > 0.1). The genotype of F_2_ plants was investigated with *GH_D13G1452-*specific primer 80Ln/80R and *Ghpericdh-*specific molecular markers 80Ln/LBSP2 and Rb2b/80R, which showed that *Ghpericdh* homozygotes accounted for 19.3%, heterozygotes accounted for 49.4%, and *GH_D13G1452* homozygotes accounted for 31.3%. The correlation coefficient between plant height and the *GH_D13G1452* gene was 0.8. Therefore, *GH_D13G1452* functioned as an incomplete dominance on plant height. The relatively low proportion of the mutant type in F_2_ might be related to the reduction of the seed germination rate caused by the *GH_D13G1452* deletion.

## Discussion

The content of ICDH was higher in roots, stems, and vascular bundles than in other tissues in other plants (Boiffin et al., 1998; Popova et al., 2002; Pascual et al., 2008). Overexpression *cytICDH* in poplar made plants stronger and higher with increased expression of genes related to vascular differentiation (Pascual et al., 2018). The *perICDH* could be activated by cadmium chloride (Romero-Puertas et al., 2006) and contributed to natural senescence (Corpas et al., 1999). However, the absence of *perICDH* does not cause abnormality in plant growth, except for stomata semi-closed in Arabidopsis (Leterrier et al., 2016). Interestingly, in addition to semi-closed stomata like Arabidopsis *pericdh*, cotton *Ghpericdh* appears more abnormal phenotypes, such as dwarf plants and developmental defect seeds, and functioned as an incomplete dominance on plant height. Additionally, the absence of *GhperICDH* made the development of seed extremely sensitive to the growth environment. *GhperICDH* was also found to be expressed with peak levels in stems and ovules of 15 days post-anthesis in upland cotton and responded to multiple external stimuli. Therefore, *GhperICDH* is indispensable in the development of stems and seeds and functions in the adaptability of cotton to the environment.

In cotton, studies mainly focus on the mapping and cloning of genes related to fibers (Mao and Cao, 2018), and the regulation mechanism of plant height is little known. Dwarf and dense cultivation of cotton can resist lodging, facilitate nutrient utilization, and mechanized operation, which could effectively improve the unit yield and reduce the labor input (Lou et al., 2021). Therefore, the study on plant dwarfing is of great significance in the high-yield breeding of cotton. Mutants are important materials for functional genomics research. The artificial mutants of wheat (Botticella et al., 2011), corn (Parry et al., 2009), tomato (Shirasawa et al., 2016), and rice (Abe et al., 2012) have accelerated their process in gene function resolving and genetic breeding. In cotton, the *GhACT17D* was found to regulate fiber elongation and plant height based on the *Li*_1_ mutant (Thyssen et al., 2017; Sun et al., 2019; Cao et al., 2021). Therefore, the cotton dwarf mutant *Ghpericdh* is useful to reveal the molecular mechanism of plant height regulation in upland cotton. More observations in cytology and physiology need to be carried out in the *Ghpericdh* mutant, and the regulation mechanism of *GhperICDH* in cotton plant height and seed development should be studied further.

## Data availability statement

The data presented in this study are deposited in the BioProject database of NCBI repository, accession number: PRJNA872936.

## Author contributions

TZ, SZ, and YC designed the experiments and wrote the manuscript. YC and WW performed the experiments. JC manipulated plant materials. All the authors have read and approved the final manuscript.
