# Supplementary material for: Deficiency of a peroxisomal NADP-isocitrate dehydrogenase leads to dwarf plant and defect seed in upland cotton
Source: Front Plant Sci. 2022 Sep 14;13:1000883. doi: 10.3389/fpls.2022.1000883 (PMC9515950; doi:10.3389/fpls.2022.1000883)
Supplement: Supplementary file 1 [file Data_Sheet_1.PDF]

Supplementary Table 1 Primers used in this study

Forward primer	Reverse primer	Application
TTCGTGCTTTTGCTGAGGCT	ACACTCTAAACATAATCGAGACTT	Specific primers for partial CDS of *GH_A13G1507*
TTCGTGCTTTTGCTGAGGCT	ACATAAAACTATCTCATCGAGAC	Specific primers for partial CDS of *GH_D13G1452*
ATGGCATTCCCTACTAAGATAAAAGT	ACATAAAACTATCTCATCGAGAC	Specific primers for complete *GH_D13G1452* CDS
GGGGTACCATGGCATTCCCTACTAAGATAAAAGT	CGGGATCCCAACTTTGGTTTCACATACATTCT	Subcellular localization for *GH_D13G1452* fused with GFP at N-terminus
ACGGGGGACGAGCTCGGTACCATGGCATTCCCTACTAAGATAAAAGT	CATGTCGACTCTAGAGGATCCCAACTTTGGTTTCACATACATTCT	Subcellular localization for *GH_D13G1452* fused with GFP at C-terminus
CCGCCACTACCGTGTTCATA	CATTGCCATCCAACCTTGCC	qPCR for *GH_D13G1452*
GAAGGCATTCCACCTGACCAAC	CTTGACCTTCTTCTTCTTGTGCTTG	qPCR for *GhUBQ7* as a control
	CGTGACTGGGAAAACCCTGGCGTT	Right border specific primer of pCAMBIA-1300 for the first round in FPNI-PCR
GTAATACGACTCACTATAGGGCACGCGTGGTNTCGASTWTSGWGTT		Fusion arbitrary degenerate primer FP1 for the first round in FPNI-PCR
GTAATACGACTCACTATAGGGCACGCGTGGTNGTAWAASGTNTSCAA		Fusion arbitrary degenerate primer FP5 for the first round in FPNI-PCR
GTAATACGACTCACTATAGGGC	CCCAACTTAATCGCCTTGCAGCACATC	Nest primers for the second round in FPNI-PCR
ACTATAGGGCACGCGTGGT	GAAGAGGCCCGCACCGATCGCCCTT	Nest primers for the third round in FPNI-PCR
TATAAGGTGGGTCCATTGTAC	CTCATGTGTTGAGCATATAAGAAACCCTTAG	*Ghpericdh* marker 80Ln/LBSP2
GAAGAGGCCCGCACCGATCGCCCTT	TGACATGCGCTAACCATCCT	*Ghpericdh* marker Rb2b/80R

Supplementary Table 2 Information on ONT sequencing of *Ghpericdh* genome.

Sample	Mean read length	Mean read quality	Median read length	Median read quality	Read length N50	Q5	Q10	Mapping rate(%)	Average depth(X)
Ghpericdh	21455.4	8.5	21417	8.9	27589	91.90%	20.00%	95.74	24.98

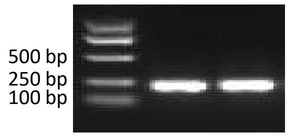

Supplementary Figure 1 The RT-PCR results of housekeeping gene GhUBQ7 in *Ghpericdh* and WT. M, DNA marker; 1, *Ghpericdh*; 2, WT.

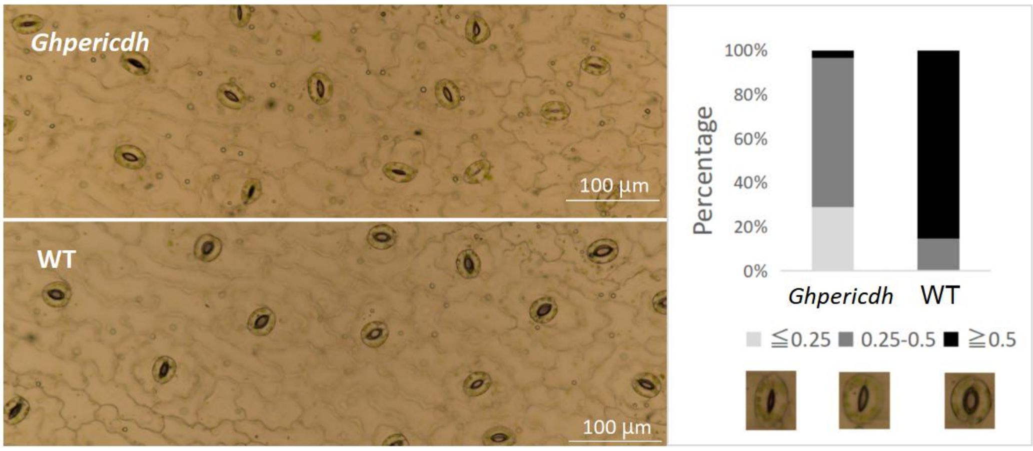

Supplementary Figure 2 The percentage of stomata with different open degree. Experiment was started in the morning after 10 hours of the dark cycle. Cotyledon detached from cotyledon-stage seedlings was floated on the incubation medium (10 mM MES, 50 mM KCl, 100µM CaCl2, pH=6.1) in Petri dishes under light condition for two hour to make stomata open fully. Lower epidermis was peeled off, transferred to a drop of incubation medium on a glass slide, and immediately observed under a microscope (Nikon Eclipse Ni, Japan) for stomata and photographed. Long axis and short axis of stomatal aperture were measured to calculate the opening degree (OD). OD=short axis/Long axis. Three individuals were randomly selected from each genotype, and three visual fields were randomly selected from each cotyledon. Most stomata of *Ghpericdh* kept semi closed (OD =0.25-0.5), while those of WT opened (OD≧0.5) under light.
